# Correction: Fernandes Prabhu et al. Integrating Artificial Intelligence, Electronic Health Records, and Wearables for Predictive, Patient-Centered Decision Support in Healthcare. *Healthcare* 2025, *13*, 2753

**DOI:** 10.3390/healthcare14081040

**Published:** 2026-04-15

**Authors:** Deepa Fernandes Prabhu, Varadraj Gurupur, Alexa Stone, Elizabeth Trader

**Affiliations:** 1 Center for Decision Support Systems and Informatics, School of Modeling and Simulation, University of Central Florida, Orlando, FL 32816, USA; 2 Center for Decision Support Systems and Informatics, School of Global Health Management and Informatics, University of Central Florida, Orlando, FL 32816, USA; varadraj.gurupur@ucf.edu; 3 EcoPreserve, 409 Fern Lake Dr, Orlando, FL 32825, USA; alexa@ecopreserve.net; 4 Center for Decision Support Systems and Informatics, School of Computer Science, University of Central Florida, Orlando, FL 32816, USA; elizabeth.trader@ucf.edu

**Error in Table**

In the original publication [1], there was a mistake in “Table 1. Existing work in artificial intelligence in healthcare” as published. The reference “Kvedar & Fogel (2016) [2]” is duplicated in Table 1. The corrected Table 1 appears below:

There was a mistake in “Table 2. Summary of existing work in electronic health record (EHR) research” as published. The years in references [27,30,35,37] do not correspond with the citations within the text. “Gurupur et al. (2025) [30]” should be changed to “Gurupur et al. (2022) [30]”. “IEEE P2933 (2021) [27]” should be changed to “IEEE P2933 (2024) [27]”. “Wright et al. (2011) [35]” should be changed to “Wright et al. (2009) [35]”. “Ratwani et al. (2018) [37]” should be changed to “Ratwani et al. (2015) [37]”. The corrected Table 2 appears below:

There was a mistake in “Table 3. Recent work in wearables and integrated health data” as published. The author name in Reference [49] and the year in Reference [48] do not match the citations within the text. “Gresham et al. (2020) [48]” should be changed to “Gresham et al. (2018) [48]”. “Muoio (2019) [49]” should be changed to “Tiase (2020) [44]”. The corrected Table 3 appears below:

**Text Correction**

There was an error in the Introduction Section, paragraph 3. The author names of reference [5] do not match the citation within the text. “Nouri-Mahdavi et al. [5]” should be changed to “Weerarathna et al. [5]”. The full paragraph should read as below:

Recent advances further underscore the growing relevance of integrated, user-centered, and predictive health systems. For example, Weerarathna et al. [5] proposed an assistive framework leveraging neural-symbolic integration to enhance human-robot collaboration in health monitoring systems. These studies highlight both the technological possibilities and critical challenges that justify our focus on completeness-aware, integrated health analytics platforms.

Due to changes in the order of the references, the citation numbers in the text have been adjusted accordingly.

**References Correction**

Reference 49 from the original manuscript had been removed.

Reference 44 in the original manuscript was cited incorrectly and has now been replaced with the following:

Tiase, V.L.; Hull, W.; McFarland, M.M.; Sward, K.A.; Del Fiol, G. Patient-Generated Health Data and Electronic Health Record Integration: A Scoping Review. *JAMIA Open* **2020**, *3*, 619–627. https://doi.org/10.1093/jamiaopen/ooaa040.

With this correction, the order of some references has been adjusted accordingly.

The authors state that the scientific conclusions are unaffected. This correction was approved by the Academic Editor. The original publication has also been updated.

## Figures and Tables

**Table 1:** Existing work in artificial intelligence in healthcare.

Study/Reference	Focus Area	Key Findings	Implications for This Work
Topol (2019) [1]	High-performance Medicine	AI augments clinical decision-making, enhances diagnostic accuracy	Supports use of AI-driven health monitoring for proactive care
Kvedar & Fogel (2016) [2]	Internet of Healthy Things	Interconnected devices enable real-time patient monitoring	Justifies integration of wearables and EHRs
ONC ISA (2023) [3]	Interoperability Standards	Highlights technical barriers in multimodal data exchange	Reinforces need for seamless EHR and wearable integration
Yin et al. (2019) [4]	Context-aware Systems	Real-world AI in healthcare often lacks contextual awareness and does not integrate patients’ lived experiences or behavioral data	Highlights the need for emotionally intelligent, context-aware, and personalized health AI
Jiang et al. (2017) [9]	AI in Healthcare	Survey of AI applications in diagnosis and risk modeling	Aligns with predictive modeling focus of this work
Blease et al. (2019) [7]	AI and Patient Trust	Clinician endorsement improves AI acceptance by patients	Justifies explainable AI features and physician–AI collaboration
Glikson & Woolley (2020) [10]	Trust in AI	Human-centered AI interaction design builds trust	Encourages provider-integrated, transparent AI systems
Zhou et al. (2019) [8]	Usability of mHealth Apps	Develops MAUQ to assess mobile health tool usability	Highlights role of user-centered design and testing
Nunes et al. (2015) [25]	Self-care Technologies	Explores tensions in digital self-care adoption	Informs need for behavior-based personalization

**Table 2:** Summary of existing work in electronic health record (EHR) research.

**Study/Reference**	**Focus Area**	**Key Findings**	**Implications for This Work**
Weiskopf and Weng (2013) [28]	Data Quality and Completeness	Defined types of missingness in EHRs and proposed metrics like Record Strength Score (RSS)	Provides baseline methods to assess data completeness
Gurupur et al. (2022) [30]	Data Quality and Completeness	Explored ontology-driven modeling of incompleteness in health data systems	Supports design of completeness-aware imputation frameworks
Mandel et al. (2016) [34]	Interoperability	Described SMART on FHIR architecture to standardize cross-system EHR access	Informs API integration for multi-source health data
IEEE P2933 (2024) [27]	Interoperability	Standardized IoT data and device interoperability for clinical use	Relevant for integrating wearable and EHR data pipelines
Chen et al. (2020) [32]	Bias and Health Disparities	Studied effects of incomplete EHRs on marginalized populations	Highlights need for equity-aware data completion methods
Wright et al. (2009) [35]	Clinical Decision Support	Demonstrated how EHR-integrated alerts can improve decision-making	Reinforces value of completeness for accurate alerts
Shortliffe and Sepúlveda (2018) [36]	Clinical Decision Support	Reviewed evolution of EHR-based decision support systems	Emphasizes integration challenges of real-time modeling
Ratwani et al. (2015) [37]	Human–Computer Interaction	Identified usability issues causing clinician fatigue	Underlines role of interface design in adoption of AI tools
Khairat et al. (2018) [38]	Human–Computer Interaction	Assessed EHR usability using objective task scores	Supports need for usability testing in AI-driven systems
Miotto et al. (2016) [39]	Machine Learning	Proposed Deep Patient, a model trained on EHRs to predict disease onset	Demonstrates feasibility of deep learning on raw EHR data
Rajkomar et al. (2018) [40]	Machine Learning	Validated scalable deep learning models for clinical prediction	Affirms predictive power of high-dimensional health data

**Table 3:** Recent work in wearables and integrated health data.

**Study/Reference**	**Focus Area**	**Key Findings**	**Implications for This Work**
Piwek et al. (2016) [42]	Adoption of consumer wearables	Widespread adoption of fitness trackers but lack of clinical integration	Highlights user engagement but underscores the need for medical validation and integration
Swan (2012) [47]	Quantified self-movement	Wearables enable self-tracking of diverse health metrics	Supports the growing need for unified health dashboards
Gresham et al. (2018) [48]	Wearables in chronic disease	Demonstrated benefits for diabetes and cardiovascular monitoring	Validates the importance of predictive analytics tools in chronic care
Tiase (2020) [44]	Integration with EHRs (Apple Health)	Data portability from consumer devices into clinical workflows	Shows the feasibility of wearable–EHR integration
Patel et al. (2012) [41]	Wearable biosensors	Review of biosensor tech for personalized health monitoring	Supports multimodal sensor fusion in the proposed tool
Haghi et al. (2017) [49]	Privacy and usability	Explores user trust and data protection in health wearables	Underlines the need for user-centered design and secure interfaces
